# Clinical similarities and differences between two large HIV cohorts in the United States and Africa

**DOI:** 10.1371/journal.pone.0262204

**Published:** 2022-04-04

**Authors:** Anne K. Monroe, Christina S. Polyak, Amanda D. Castel, Allahna L. Esber, Morgan E. Byrne, Jonah Maswai, John Owuoth, Lucas Maganga, Emmanuel Bahemana, Yakubu Adamu, Michael Iroezindu, Hannah Kibuuka, Francis Kiweewa, Alan E. Greenberg, Trevor A. Crowell, Julie A. Ake, on behalf of the DC Cohort Executive Committee and AFRICOS Study Group

**Affiliations:** 1 Department of Epidemiology and Biostatistics, Milken Institute School of Public Health at the George Washington University, Washington DC, United States of America; 2 U.S. Military HIV Research Program, Walter Reed Army Institute of Research, Silver Spring, Maryland, United States of America; 3 Henry M. Jackson Foundation for the Advancement of Military Medicine, Bethesda, Maryland, United States of America; 4 U.S. Army Medical Research Directorate – Africa, Kericho, Kenya; 5 U.S. Army Medical Research Directorate – Africa, Kisumu, Kenya; 6 HJF Medical Research International, Kisumu, Kenya; 7 National Institute for Medical Research - Mbeya Medical Research Centre, Mbeya, Tanzania; 8 HJF Medical Research International, Mbeya, Tanzania; 9 U.S. Army Medical Research Directorate – Africa, Abuja, Nigeria; 10 HJF Medical Research International, Abuja, Nigeria; 11 Makerere University-Walter Reed Project, Kampala, Uganda; Mater Misericordiae University Hospital, IRELAND

## Abstract

**Background:**

Washington, DC, and sub-Saharan Africa are both affected by generalized HIV epidemics. However, care for persons living with HIV (PLWH) and clinical outcomes may differ in these geographically and culturally diverse areas. We compared patient and clinical site characteristics among adult persons living with HIV (PLWH) enrolled in two longitudinal HIV cohort studies—the African Cohort Study (AFRICOS) and the DC Cohort.

**Methods:**

The DC Cohort is a clinic-based city-wide longitudinal cohort comprised of PLWH attending 15 HIV clinics in Washington, DC. Patients’ socio-demographic characteristics, clinical evaluations, and laboratory data are retrospectively collected from electronic medical records and limited manual chart abstraction. AFRICOS is a prospective observational cohort of PLWH and uninfected volunteers attending 12 select HIV care and treatment facilities in Nigeria, Kenya, Uganda and Tanzania. AFRICOS study participants are a subset of clinic patients who complete protocol-specific visits every 6 months with history and physical examination, questionnaire administration, and blood/sputum collection for ascertainment of HIV outcomes and comorbidities, and neurocognitive and functional assessments. Among participants aged ≥ 18 years, we generated descriptive statistics for demographic and clinical characteristics at enrollment and follow up and compared them using bivariable analyses.

**Results:**

The study sample included 2,774 AFRICOS and 8,420 DC Cohort participants who enrolled from January 2013 (AFRICOS)/January 2011 (DC Cohort) through March 2018. AFRICOS participants were significantly more likely to be women (58.8% vs 27.1%) and younger (83.3% vs 61.1% aged < 50 years old) and significantly less likely to be MSM (only 0.1% of AFRICOS population reported MSM risk factor) than DC Cohort. Similar rates of current viral suppression (about 75% of both samples), hypertension, hepatitis B coinfection and alcohol use were observed. However, AFRICOS participants had significantly higher rates of CD4<200 and tuberculosis and significantly lower rates of obesity, DM, hepatitis C coinfection and syphilis.

**Conclusions:**

With similar viral suppression outcomes, but many differences between our cohorts noted, the combined sample provides unique opportunities to assess and compare HIV care and treatment outcomes in the U.S. and sub-Saharan Africa. Comparing these two cohorts may inform care and treatment practices and may pave the way for future pathophysiologic analyses.

## Introduction

There is a generalized HIV epidemic in many African countries, with an HIV prevalence of 6.8% in East and Southern Africa and 2.8% in Nigeria [1–3]. Additionally, there are concentrated subepidemics in key populations in Africa, including men who have sex with men (MSM) and transgender women [4]. The U.S. does not have a generalized HIV epidemic, however there are areas of the U.S. with high HIV prevalence, including Washington DC, which has an overall HIV prevalence of 1.9% [5]. Because HIV disproportionately affects members of racial and ethnic minorities, the HIV prevalence in Washington DC is much higher when considering only Black or Latino males, at 4.4% and 2.1%, respectively [5].

Eliminating HIV in a community is possible only with both treatment of people living with HIV (PLWH) and prevention of HIV transmission. This strategy has been ongoing in both the U.S. and Africa, with some similar goals, such as medication adherence, and distinctive challenges based on context. Both internationally [6] and locally [7] there are goals for meeting HIV care continuum outcomes, aiming for 90% of individuals in care to be being on antiretroviral therapy (ART) and 90% of people on therapy suppressed. To achieve these goals, similar strategies are necessary including emphasis on linkage to care, retention in care, and viral suppression among PLWH. While the strategies are similar, however, resources may differ. Heavily resourced areas with a variety of care venues and ART options may have different outcomes than those with centralized treatment distribution and fewer ART options. Additionally, comorbid conditions increasingly contribute to mortality in the context of well-controlled HIV, therefore describing variations in comorbidities and comorbidity management may be informative. Approaches to managing both HIV and other comorbidities may differ based on local context; however, there may be lessons learned from comparing these settings.

With these principles in mind, in 2017 we initiated a collaboration between the African Cohort Study (AFRICOS) and the DC Cohort, two longitudinal HIV cohort studies enrolling patients across different clinical settings. These cohorts have similar goals and are run in settings where HIV is generalized, but in different clinical and cultural settings that may lead to differences in clinical outcomes among PLWH between these cohorts. The long-term goals of this collaboration are to generate large datasets with geographically diverse participants that will allow us to design inquiries that leverage the strengths of the two cohorts and identify opportunities to compare outcomes in meaningful ways to address high-priority research questions. As an initial step, the objective of this analysis was to compare participant characteristics, clinical site characteristics, treatment response and comorbidities across the two cohorts.

## Methods

### AFRICOS study population and data collection

AFRICOS is a long term prospective observational HIV-focused cohort started in January 2013. Inclusion criteria for the study include adults aged 18 and older living with and at risk for HIV receiving care at HIV care and treatment facilities supported by the U.S. Military HIV Research Program (MHRP) and U.S. President’s Emergency Plan for AIDS Relief (PEPFAR) in Kenya, Nigeria, Tanzania and Uganda. Study participants provide written informed consent, and the study was approved by the institutional review boards of the Walter Reed Army Institute of Research, Makerere University School of Public Health, Kenya Medical Research Institute, Tanzania National Institute of Medical Research, and Nigerian Ministry of Defence. At the time of this analysis, target enrollment for AFRICOS was 3,000 PLWH and 600 people without HIV. The study serves as an evaluation tool for the MHRP PEPFAR program and also facilitates investigation into HIV comorbidities and pathogenesis in an African context. Study participants are drawn from health facility clinic patient populations, allowing MHRP to monitor the impact of HIV-directed health and preventive services that fall under national guidelines. Study participants complete visits every 6 months during which they undergo a medical history, physical examination, brief neurocognitive battery, functional assessment, depression screen, questionnaire administration and collection of blood and sputum. HIV outcomes, infectious comorbidities, and non-infectious comorbidities are assessed. For AFRICOS, data from participants with HIV from enrollment and the most recent visit up to December 15, 2017 were included.

For demographic and substance use characteristics within AFRICOS, all variables were collected by self-report. For HIV laboratory parameters, viral load and CD4 were collected at the enrollment and most recent study visits [8].

Multiple comorbid conditions were assessed using both laboratory and clinical diagnosis data. These conditions included elevated blood pressure, hypercholesterolemia, non-fasting dysglycemia, renal insufficiency, tuberculosis, hepatitis B, hepatitis C, syphilis. S1 Table provides additional detail on how comorbidities were defined in AFRICOS and the DC Cohort.

AFRICOS has site-related information about size of clinic, type of clinic, and types of providers at each clinic collected from the Principal Investigators at each site using standard questions. AFRICOS procedures have been described in detail previously [9].

### DC cohort study population and data collection

The DC Cohort is a clinic-based city-wide longitudinal cohort that enrolls HIV-infected participants at 15 HIV clinics in Washington, DC, started in January 2011 without restriction on age or other characteristics. Written informed consent is provided by study participants, and the study was approved by the George Washington University Institutional Review Board and the Institutional Review Boards of participating sites, as required. Patients’ socio-demographic, HIV transmission risk factors, and HIV/AIDS diagnosis dates are collected from electronic medical records (EMRs) and supplemented with manual abstraction of historical data as needed. Additionally, information on clinical encounters, diagnoses of comorbidities including hepatitis, treatments (antiretroviral therapy and other treatments), and laboratory tests including CD4 cell counts, HIV RNA, and resistance testing is collected from all participants. The frequency of specific evaluations is dictated by provider discretion according to the standard of care at each clinical site. An additional unique feature of the DC Cohort is that data are linked to District of Columbia Department of Health surveillance data on HIV and sexually transmitted infections. DC Cohort procedures have been described in additional detail previously [10, 11].

For DC Cohort, data from enrollment and laboratory data up to March 31, 2018 were included. All demographic and substance use characteristics were collected via chart abstraction. For enrollment HIV laboratory parameters, viral load and CD4 were abstracted from the medical record within 6 months prior to consent or up to 1 month after consent.

Multiple comorbid conditions were assessed using laboratory data, medical record abstraction, and billing codes. For example, the diagnosis of chronic Hepatitis C in the DC Cohort is based on a chronic HCV diagnosis in the EMR. In the absence of laboratory data, participants were considered negative for that condition (See S1 Table).

Finally, site level characteristics in the DC Cohort were assessed using a site assessment survey that was undertaken at all DC cohort sites in the first quarter of 2017. Site principal investigators (PIs) received an electronic survey including information about care delivery at the clinic within the following domains: types of medical providers, types of medical services offered, availability of wraparound services such as navigation services.

### Analytic methods

Data for the two cohorts were analyzed independently, meaning that different analysts conducted the analysis with each cohort. Quality control was assured by joint design of the analytic plan, use of agreed-upon definitions and review of analytic code by each study group. These analyses used descriptive statistics (including frequencies, measures of central tendency, and ranges) and the calculation of prevalence of comorbidities. Missing data were not imputed. Univariate analysis using Pearson chi-squared and Fisher’s exact test for categorical variables and Wilcoxon or independent samples T-test were used for continuous variables. SAS version 9.4 (SAS Institute, Cary NC) was used by both analysts; additionally, Stata version 15 (StataCorp LP, College Station, TX) was used by the AFRICOS analyst. This study was designed to be primarily descriptive. The chi-square tests are exploratory in nature and not based on specific pre-specified hypotheses. Therefore, power calculations were not conducted. However, the confidence intervals do help convey the precision of the relative risk estimates.

## Results

**Fig 1:**
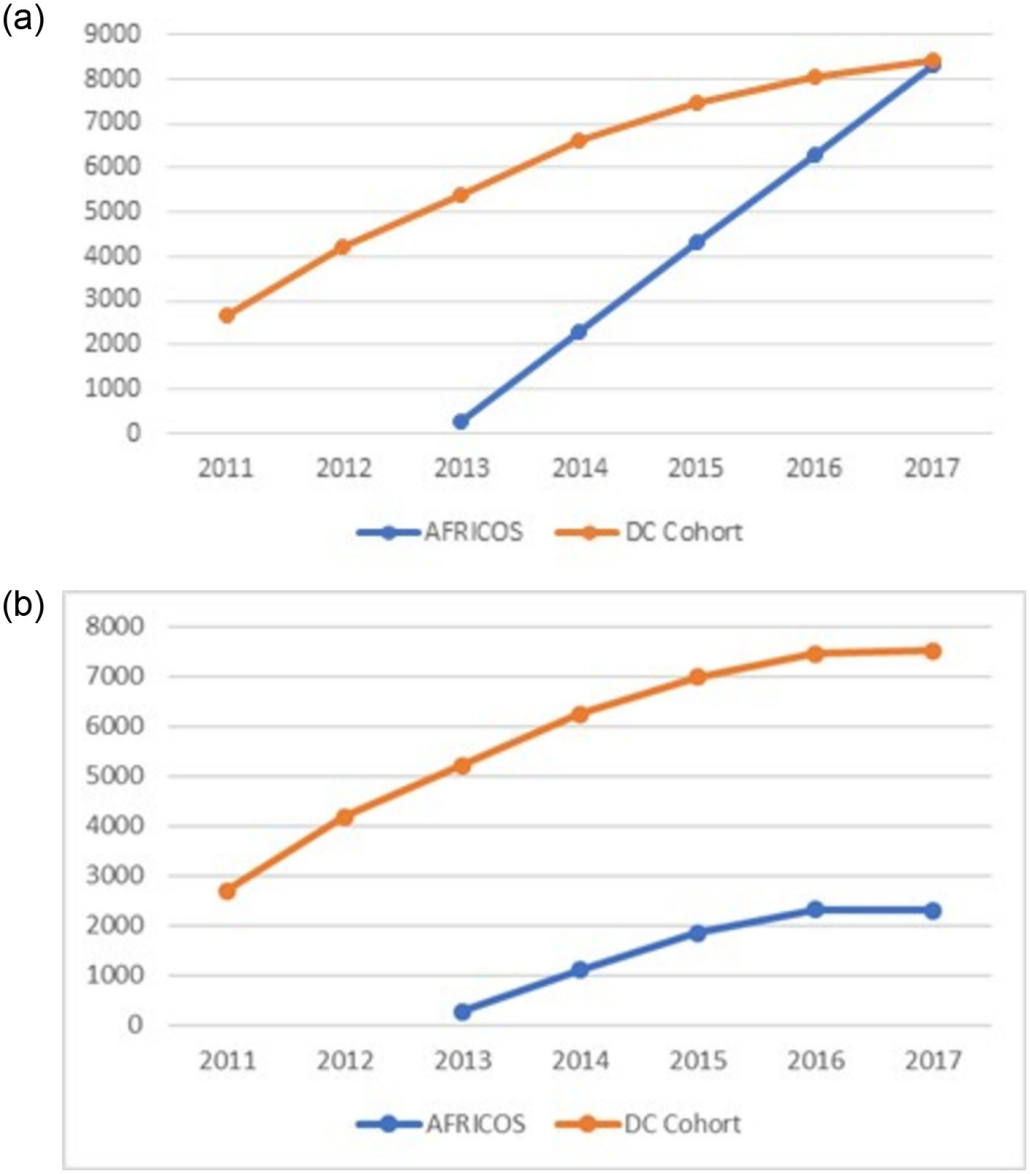
a. Cumulative enrollment, 2011–2017, AFRICOS and DC Cohort. b. Active participants per year, 2011–2016, AFRICOS and DC Cohort.

Fig 1a and 1b display the cumulative enrollment and the active participants per year in AFRICOS and the DC Cohort.

Enrollment demographic characteristics of participants in the two cohorts are shown in Table 1. Overall, 2,774 AFRICOS (enrolled in 2013–17) and 8,420 DC Cohort (enrolled in 2011–18) participants were included in this analysis. Notable differences between the two groups were that more AFRICOS participants were female compared with DC Cohort participants (58.8% vs 27.1%, p<0.0001) and that more AFRICOS participants were <50 years old (83.3% vs 61.1%, p<0.0001). The majority of patients in DC cohort were 50 or older (40.9%) while the majority in AFRICOS were 25–39 years old (47.1%). In total, there were 5,782 person-years of observation time from AFRICOS and over 32,000 person-years from DC Cohort, including median 2.1 (IQR 1.1–3.1) years per participant from AFRICOS and 3.8 (IQR 2.3–5.7) years for participant for DC Cohort. Most participants were still active at the end of the observation period (92.0% and 71.9% for AFRICOS and DC Cohort respectively, p<0.0001). Overall, there was a lower proportion of deaths among AFRICOS participants (3.2% vs. 5.1%, p = 0.02), however, length of follow up was not accounted for in the analysis. Additionally, a lower proportion of AFRICOS participants either transferred care to another clinic or were lost to follow up (4.8% vs 23.6%, p<0.0001).

**Table 1:** Enrollment participant characteristics by cohort.

	DC Cohort (N = 8420) N (%)	AFRICOS (N = 2774) N (%)	p-value***
**Gender**			<.0001
Male	5981 (71)	1144 (41)	
Female	2282 (27)	1630 (59)	
Transgender	157 (2)	--	
**Age (years)**			<.0001
18–24	448 (5)	223 (8)	
25–39	2147 (26)	1306 (47)	
40–49	2379 (28)	782 (28)	
50+	3446 (41)	463 (17)	
**Employed**			0.0006
Yes	2473 (44)	1110 (40)	
No	3154 (56)	1664 (60)	
**Race**			
Non-Hispanic Black	6503 (77)	2774 (100)	<0.0001
Non-Hispanic White	1080 (13)	0 (0)	
Hispanic	463 (6)	0 (0)	
Other	157 (2)	0 (0)	
Unknown	217 (3)	0 (0)	
**Alcohol use**			0.92
Yes	1182 (19)	530 (19)	
No	5039 (81)	2244 (81)	
**Tobacco use**			<.0001
Yes	3132 (42)	128 (5)	
No	4289 (58)	2645 (95)	
**Injection drug use**			<.0001
Yes	674 (8)	3 (0)	
No	7746 (92)	2771 (100)	
**Sexual behavior**			<.0001
MSM*	3298 (39)	3 (0)	
Heterosexual male	2837 (34)	1141 (41)	
Heterosexual female	2282 (27)	1630 (59)	

Note:

*MSM defined as males who reported having a regular and/or casual male partner or males who have reported having anal receptive intercourse.;

** Data as of Dec. 15, 2017 for AFRICOS and Mar. 31, 2018 for DC Cohort

Abbreviations: MTF Male to female; FTM Female to male

Table 2 displays the HIV-related characteristics of the study participants. As compared to participants in the DC Cohort, more AFRICOS participants had nadir CD4 <200 cells/mm^3^ (45.7% vs 34.4%, p<0.0001) and fewer had most recent CD4 ≥500 cells/mm^3^ (45.1% vs 58.2%%, p<0.0001). AFRICOS participants were less likely to be on ART at enrollment (68.1% vs. 95.1%, p<0.0001), potentially contributing to other clinical differences. Among participants who were on ART at enrollment, AFRICOS participants were less often taking tenofovir (58.7% vs 76.9%, p<0.0001) and more often taking non-nucleoside reverse transcriptase inhibitors (NNRTIs) (61.0% vs 36.4%). No AFRICOS participants took integrase inhibitors (INSTIs) compared with 32.8% of DC Cohort participants (p<0.0001). The proportion of participants with HIV RNA <50 copies/mL at last assessment was very similar (75.2% in AFRICOS vs 76.9% in DC Cohort, p<0.0001). The majority of participants in both cohorts were on ART at the time of last assessment (95.8% of AFRICOS participants and 98.7% of DC Cohort participants).

**Table 2:** HIV-related characteristics by cohort.

	DC Cohort (N = 8420)	AFRICOS (N = 2774)	p-value
**Years since HIV diagnosis, mean(SD)** *	5.15 (6.2)	4.7 (3.6)	
**On ART at enrollment** *			<0.0001
Yes	7744 (92)	1890 (68)	
No	676 (8)	884 (32)	
**CD4 nadir (cells/mm**^**3**^**)** *			<0.0001
500 +	1702 (19)	253 (9)	
350–499	1565 (18)	257 (9)	
200–349	2087 (24)	600 (22)	
<200	3048 (34)	1268 (46)	
Missing/unknown	447 (5)	396 (14)	
**Enrollment CD4 (cells/mm** ^**3**^ **)** *			<0.0001
500 +	4347 (52)	918 (33)	
350–499	1585 (19)	613 (22)	
200–349	1176 (14)	680 (25)	
<200	838 (10)	548 (20)	
Missing/unknown	474 (6)	15 (1)	
**Most recent CD4 (cells/mm** ^**3**^ **)** **			<0.0001
500 +	4898 (58)	1237 (45)	
350–499	1375 (16)	678 (25)	
200–349	970 (12)	500 (18)	
<200	703 (8)	327 (12)	
Missing/unknown	474 (6)	3 (0)	
**Enrollment viral load (copies/mL)** *			<0.0001
<50	5558 (66)	1376 (50)	
50+	2390 (28)	1398 (50)	
**Most recent viral load (copies/mL)** **			<0.0001
<50	6110 (77)	2087 (75)	
50+	1838 (23)	687 (25)	
Median (IQR)	10 (10–43)	U† (U-49.0)	
Enrollment viral load suppressed (<200 copies/mL)			
Yes	6267 (79)	951 (55)	
Most recent viral load suppressed (< 200 copies/mL)			
Yes	6725 (85)	2221 (80)	
**Among Participants on ART at enrollment** *			
On ART containing tenofovir	6264 (74)	1110 (59)	<0.0001
On ART containing efavirenz	1961 (23)	1005 (53)	<0.0001
On ART containing nevirapine	121 (1)	677 (36)	<0.0001
On ART containing a non-nucleoside reverse transciptase inhibitor	2969 (35)	1692 (61)	<0.0001
On ART containing a protease inhibitor	3490 (41)	120 (6)	<0.0001
On ART containing a integrase inhibitor	2668 (32)	0 (0.0)	<0.0001

Note:

* Data collected at enrollment,

**Data collected at most recent visit

*** ART start date not accurately captured

^†^ U Undetectable (below the limit of detection of assay)

**Fig 2:**
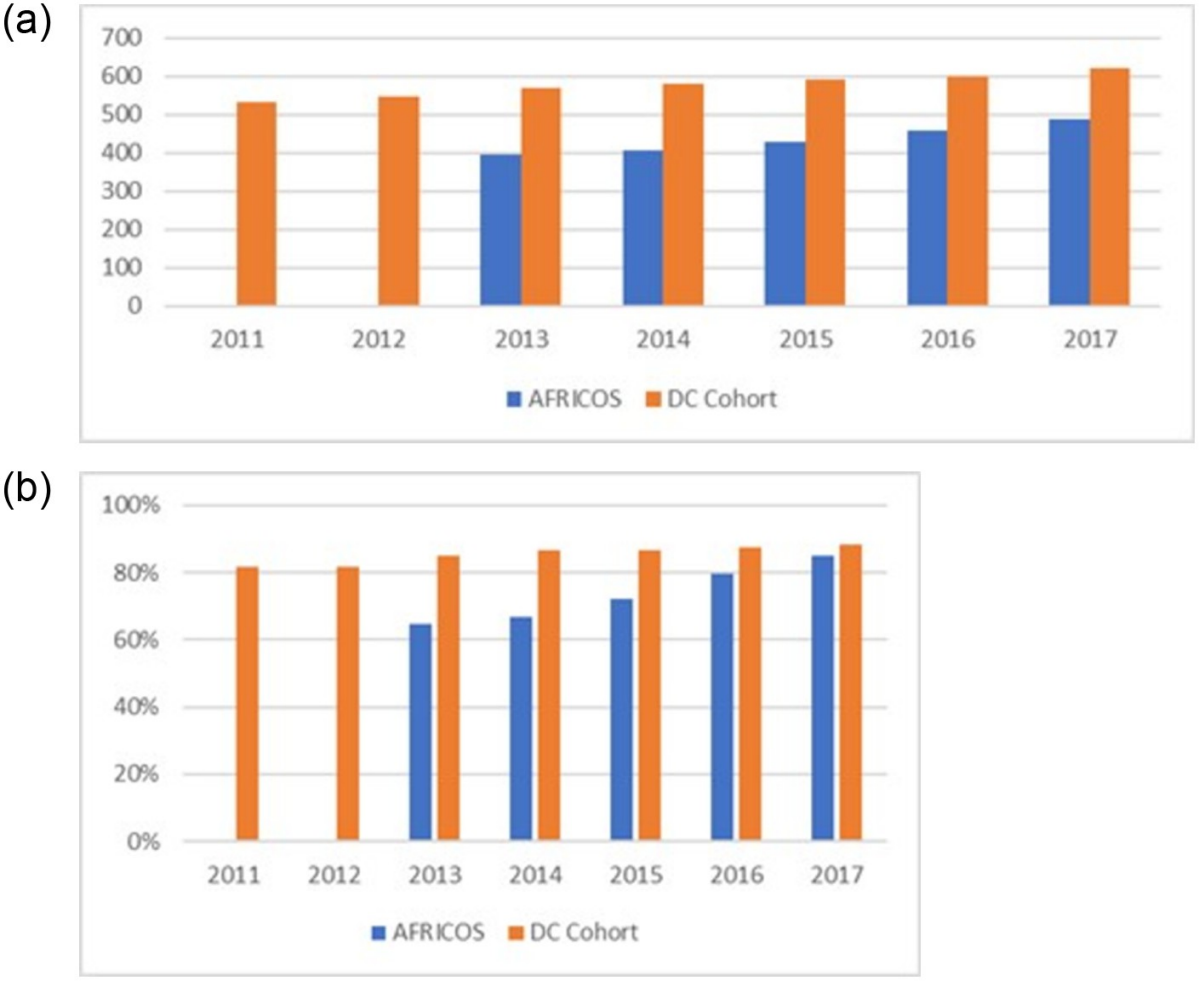
a. Median CD4 count by year, 2011–2017, AFRICOS and DC Cohort. b. Proportion of participants with suppressed HIV RNA by year, 2011–2017, AFRICOS and DC Cohort.

Fig 2a shows the *Median CD4 count by year, 2011–2017, for AFRICOS and DC Cohort*. Fig 2b. *shows the proportion of participants with suppressed HIV RNA by year, 2011–2017, AFRICOS and DC Cohort*.

Shown in Table 3 are the co-infections and comorbidities by cohort (comorbidity definitions in S1 Table). The prevalence of hypercholesterolemia was higher in AFRICOS participants, (36.5% vs 21.2%, p<0.0001) however prevalence of blood glucose >199 mg/dL (2.2% vs 7.8%, p<0.0001), and prevalence of glomerular filtration rate by Modification of Diet in Renal Disease Study equation < 60 mL/min/1.73 m^2^ (4.8% vs 6.8%, p = 0.02) were both lower. Obesity with body mass index >30 kg/m^2^ was lower in AFRICOS participants (6.7% vs. 27.6%, p<0.0001).

**Table 3:** Co-infections and comorbidities by cohort, at enrollment and most recent visit.

	DC Cohort (N = 8420 enrollment)	AFRICOS (N = 2774 enrollment)	p-value
**BMI at enrollment**			<0.0001
<18.5	356 (4)	304 (11)	
18.5–24.99	2986 (36)	1757 (63)	
25–29.99	2749 (33)	527 (19)	
30+	2323 (28)	186 (7)	
**BMI at most recent**			
<18.5	336 (4)	264 (10)	<0.0001
18.5–24.99	2552 (30)	1706 (62)	
25–29.99	2538 (30)	554 (20)	
30+	2989 (36)	250 (9)	
**Elevated blood pressure (enrollment)**	3024 (36)	352 (36)	<0.0001
**Elevated blood pressure (most recent)**	2077 (25)	430 (16)	<0.0001
**Hypercholesterolemia (enrollment)**	1781 (21)	517 (18)	<0.0001
**Hypercholesterolemia (most recent)**	1579 (19)	634 (23)	<0.0001
**Non-Fasting Dysglycemia (enrollment)**	660 (8)	272 (10)	<0.0001
**Non-Fasting Dysglycemia (most recent)**	432 (5)	297 (11)	<0.0001
**Renal insufficiency (enrollment)**	571 (7)	38 (1)	0.015755
**Renal insufficiency (most recent)**	635 (8)	36 (1)	<0.0001
**Anemia (enrollment)**	1076 (13)	1067 (39)	<0.0001
**Anemia (most recent)**	1144 (14)	1038 (38)	<0.0001
**Tuberculosis (enrollment)**	13 (0)	97 (3)	<0.0001
**Tuberculosis (most recent)**	16 (0)	31 (1)	<0.0001
**Hepatitis B (enrollment)**	315 (4)	73 (3)	0.61012
**Hepatitis B (most recent)**	181 (2)	74 (3)	0.10
**Hepatitis C (enrollment)**	1180 (14)	25 (1)	<0.0001
**Hepatitis C (most recent)**	586 (7)	--	--
**Syphilis (enrollment)**	1996 (24)	150 (5)	<0.0001
**Syphilis (most recent)**	1406 (17)	92 (5)	<0.0001

Note: Elevated Blood Pressure: Systolic BP>139 or Diastolic BP>89 or on hypertension medications; Hypercholesterolemia: Cholesterol>199 or on cholesterol (lipid-lowering) medications; Non-Fasting Dysglycemia: glucose >199 or on glucose-lowering medications; Renal Insufficiency: GFR<60 by MDRD Study equation; Anemia was defined as hemoglobin <12 g/dL for males or <13 g/dL for females; Tuberculosis: Positive Gene Xpert or on a full course of TB treatment in the absence of bacteriological confirmation (AFRICOS); history of TB (AFRICOS). Hepatitis B: Reactive Hepatitis B Surface Antigen + confirmatory test (AFRICOS); chronic Hepatitis B diagnosis (DC Cohort); Hepatitis C: Reactive Hepatitis C Virus Antibody + confirmatory (AFRICOS); chronic Hepatitis C diagnosis (DC Cohort) Syphilis: Reactive RPR or VDRL (AFRICOS); Positive non treponemal test [RPR/VDRL] up to 1 year after enrollment or Syphilis diagnosis code before or up to 30 days after enrollment (DC Cohort)

Finally, Table 4 presents the site-level characteristics of each cohort. All AFRICOS sites are large (>500 patients), while DC Cohort clinics vary in size, with over half being large, one-third medium sized (200–500 patients) and the others small (<200 patients). All AFRICOS sites are hospital-based, while DC Cohort has a mix of hospital and community-based sites. All AFRICOS sites have primary care services, HIV counseling and testing, adherence support, and an on-site clinical pharmacy. Most DC Cohort sites (80.0%) offer adherence support services and 66.7% have an on-site clinical pharmacy.

**Table 4:** Site level characteristics by cohort.

	DC Cohort (N = 15)	AFRICOS (N = 12)
	N (%)	N (%)
**Clinic size**		
Small (<200)	2 (13%)	0
Medium (200–500)	5 (33%)	0
Large (>500)	8 (53%)	12 (100%)
**Level of care**		
Primary/outpatient/community based clinic	6 (40%)	0
Secondary/hospital based clinic	9 (60%)	12 (100%)
**Number of providers per site, range**		
Medical Doctors	1,10	0,6
Fellows	1,5	0
Physician Assistants/Clinical Officers	1,5	0,7
NPs	1,4	0
RNs	2,6	3,9
**Available interventions and services**		
Primary care	15 (100)%	100%
HIV Counseling and Testing	15 (100%)	100%
Adherence support	12 (80%)	100%
On site clinical pharmacy	10 (67%)	100%

## Discussion

This analysis marks the start of a collaboration to explore similarities, differences and research synergies between two large HIV-focused cohorts in Sub-Saharan Africa and Washington, DC. This initial comparison of the cohorts revealed several differences in the demographic characteristics of the study populations. However, in both cohorts, the proportion of individuals achieving viral suppression was below the UNAIDS 90-90-90 target of 90% viral suppression, with numbers closer to 75%. These cohorts represent in-care populations, therefore interventions to enhance medication access and/or adherence may be necessary to help participants in both cohorts achieve viral suppression and meet the 90% suppressed target.

Demographic differences observed between the two cohort studies reflect the demographics of HIV in Africa and in Washington, DC. In Sub-Saharan Africa, the epidemic is driven by heterosexual transmission, women make up the majority of PLWH, and youth are disproportionately affected by new HIV infections [2]. In contrast, the DC Cohort study population reflects the aging population of PLWH in the US, partially due to decreasing incidence of new infections. The population most impacted by in DC is Black men, with an HIV prevalence among of 4.4%, far above the general population prevalence of 1.9% [5].

Although the epidemics differ, the goal of achieving optimal HIV outcomes is the same. Full achievement of 90-90-90 requires identifying individuals who are not virally suppressed in near real-time and tailoring interventions to meet their need. The DC Cohort has developed a Dashboard so that individuals who are not suppressed can be identified and outreach can be performed. For MHRP-supported PEPFAR clinics with unsuppressed patients, enhanced adherence counseling has been implemented and all participants with VL >1000 c/mL are enrolled.

We found that AFRICOS participants had a lower nadir CD4 count at enrollment than did DC Cohort participants. WHO HIV treatment guidelines have shifted over time from treating only people with a CD4 count cell count less than 200 cells/mm^3^ to treating all individuals with HIV; however, late initiation of ART is still a problem [12]. In both the U.S. and sub-Saharan African countries, both delays in HIV diagnosis [13] and delayed entry to HIV care [14] may impact baseline CD4 and subsequent CD4 recovery. The global trend since 2010 has been that ART is being initiated at a higher CD4 count, although the majority of individuals still start at a CD4 below 350 cells/mm^3^ [15]. Scaling up HIV testing and treatment, within the framework of Ending the HIV Epidemic efforts in the US and PEPFAR and other initiatives in Sub-Saharan Africa, can promote earlier diagnosis and treatment. There was a small difference in the proportion of individuals who were virally suppressed comparing AFRICOS and DC Cohort. The reported rate of viral suppression among those on ART in East and Sub-Saharan Africa is 79% suppressed [3]. In the U.S., the most recent estimate of viral suppression nationally from the CDC was 62% [16]. Our sample in the DC Cohort represents a sample of individuals engaged in HIV care, which is why our estimates are likely higher than those for the general U.S. population.

Both Department of Health and Human Services (DHHS) and WHO guidelines support using antiretroviral therapy in all PLWH, with WHO guidelines noting that priority consideration for ART should be given to those with advanced clinical disease or CD4 ≦350 cells/mm^3^ [17]. In the WHO guidelines, recommended first line therapy is an NNRTI, usually efavirenz, although dolutegravir is also recommended when it is available. Prior work in the AFRICOS study has demonstrated that time to ART initiation is shortening [12]. Treatment guidelines in the U.S. from the DHHS recommend integrase inhibitor therapy for first-line treatment of HIV [18]. This is typically given in combination with the NRTIs with the least potential for metabolic toxicity. In our sample, no one from AFRICOS was on an integrase inhibitor, while 32% of the DC Cohort sample was on an integrase inhibitor at baseline. Although the data are not shown, the proportion of DC Cohort participants receiving integrase is even higher when considering current regimen.

The burden of metabolic comorbidities in the U.S. is increasing with an aging population of PLWH. In prior work estimating the burden of metabolic complications in the DC Cohort, we found a high burden of hypertension (50%), diabetes (13%), dyslipidemia (48%) and obesity (35%) [19]. The prevalence estimates in that article vary somewhat from those presented here due to differences in timeframes and definitions. A systematic review and meta-analysis of metabolic comorbidities in low income countries, predominately in Sub-Saharan Africa, revealed a high burden of comorbidities (hypertension 21%, hypercholesterolemia 22%, obesity 8% and diabetes 1.3–18%) [20]. This is a major challenge to prolonging life with HIV in the setting of durable viral suppression. Although the proportions were statistically significantly different, the proportions of individuals in DC Cohort and AFRICOS with elevated blood pressure and non-fasting dysglycemia at enrollment were similar. Much higher proportion of AFRICOS participants had anemia. Renal insufficiency was higher in the DC Cohort, likely representing older age and/or longer time on ART. Although our analysis did not directly examine improvement in medical comorbidities in individual participants over time, a lower proportion of individuals in both cohorts had elevated blood pressure on their most recent clinical assessment than at enrollment. Metabolic comorbidities were not uniformly improved however. Participation in HIV care can be an opportunity to engage in care for other comorbidities, improving overall health outcomes.

When considering site characteristics, the variety of clinical sites in the DC Cohort reflects the variety of venues providing HIV care in the U.S. There is ongoing work within the DC Cohort to determine which features of clinical sites are most strongly associated with improved HIV outcomes. Within AFRICOS, there is less variability between the clinic types, although all provide wraparound services such as enhanced adherence services. There may also be some drawbacks to the central provision of services, because it limits patient choice and any disruption to provision of services to that site disrupts services throughout the area.

A major strength of this study is the large, diverse samples of patients and the breadth of information available. A limitation of the data presented is that calculated p-values should be interpreted within the context of the large sample size and the multiple comparisons made. Additional limitations include that there is an element of retrospective data abstraction for historic data within DC cohort while there is prospective data collection only in AFRICOS. There are limits on interpretation of cohort differences given that AFRICOS comprises four different national settings and larger group of clinics which could lead to greater variation within the AFRICOS cohort than DC Cohort.

In conclusion, there are similar HIV care continuum goals for participants in these two cohorts and the proportion of people virally suppressed is not yet at goal. However, time to ART initiation in AFRICOS is shortening and the proportion of individuals virally suppressed increased over time. Interventions to reach goal will need to be locally tailored based on the individuals most in need of extra support in both venues. We see great potential for this collaboration, particularly given the large number of participants in the two cohorts and the variety of variables collected. Possible future projects may include investigations to examine: outcomes of HIV treatment regimens over time, the extent and impact of co-morbidities, and factors associated with retention in HIV care across settings. Our ultimate goal is to focus on identifying new approaches to enhancing HIV care delivery in a wide variety of clinical sites.

## Supporting information

S1 Table Comorbidity definitions. (DOCX)
