# Supplementary material for: Clinical similarities and differences between two large HIV cohorts in the United States and Africa
Source: PLoS One. 2022 Apr 4;17(4):e0262204. doi: 10.1371/journal.pone.0262204 (PMC8979457; doi:10.1371/journal.pone.0262204)
Supplement: S1 Table — (DOCX) [file pone.0262204.s001.docx]

**S1 Table. Comorbidity definitions.**

| Condition | Definition | Timeframe |
| --- | --- | --- |
| Elevated blood pressure | Systolic Blood Pressure > 139 mmHg OR Diastolic Blood Pressure >89 mmHg OR on medication for elevated blood pressure | AFRICOS: at enrollment  DC Cohort: Lab value within 1 year after enrollment [date closest to enrollment] |
| Hypercholesterolemia | Cholesterol>199 mg/dL OR on medication for hypercholesterolemia | AFRICOS: at enrollment  DC Cohort:  Lab value within 1 year after enrollment [date closest to enrollment] OR Lipid-lowering medication within 1 year of enrollment [drug start date may be prior to enrollment] |
| Non-fasting dysglycemia | Glucose >199 mg/dL OR on medication for hyperglycemia | AFRICOS: at enrollment  DC Cohort:  Lab value within 1 year after enrollment [date closest to enrollment] OR Lipid-lowering medication within 1 year of enrollment [drug start date may be prior to enrollment] |
| Renal insufficiency | GFR by Modification of Diet in Renal Disease Study equation < 60 mL/min/1.73 m² | AFRICOS: at enrollment  DC Cohort:  Lab value within 1 year after enrollment [date closest to enrollment] |
| Anemia | Hemoglobin <12 g/dL for males or <13 g/dL for females | AFRICOS: at enrollment  DC Cohort:  Lab value within 1 year after enrollment [date closest to enrollment] |
| Tuberculosis (TB) | Active TB (AFRICOS); history of TB (DC Cohort) | AFRICOS: labs collected at enrollment  DC Cohort: History of TB at enrollment: ICD 9 or ICD 10 diagnosis code indicating history of TB in baseline HIV data table. |
| Hepatitis B (HBV) | Reactive Hepatitis B surface Antigen + confirmatory test (AFRICOS); Chronic HBV diagnosis in EMR (DC Cohort) | AFRICOS: labs collected at enrollment  DC Cohort: ICD 9 or 10 code indicating chronic HBV diagnosis code before or up to 30 days after enrollment |
| Hepatitis C (HCV) | Reactive Hepatitis C Virus Antibody + confirmatory (AFRICOS); Chronic HCV diagnosis in EMR (DC Cohort) | AFRICOS: labs collected at enrollment  DC Cohort: ICD 9 or 10 code indicating chronic HCV diagnosis code before or up to 30 days after enrollment |
| Syphilis | Reactive RPR or VDRL (AFRICOS); Reactive RPR or VDRL OR Syphilis diagnosis code in EMR before or up to 30 days after enrollment (DC Cohort) | AFRICOS: labs collected at enrollment  DC Cohort:  Positive non treponemal test [RPR/VDRL] up to 1 year after enrollment OR syphilis diagnosis code before or up to 30 days after enrollment |
